# An RNA-Seq analysis of coronavirus in the skin of the Pangolin

**DOI:** 10.1038/s41598-024-51261-x

**Published:** 2024-01-09

**Authors:** Siwei Deng, Xuechen Tian, Robert Belshaw, Jinfeng Zhou, Siyuan Zhang, Yixin Yang, Chang Huang, Weikang Chen, Hailu Qiu, Siew Woh Choo

**Affiliations:** 1 Department of Biology, College of Science, Mathematics and Technology, Wenzhou-Kean University, 88 Daxue Road, Ouhai, Wenzhou, Zhejiang 325060 China; 2 Zhejiang Bioinformatics International Science and Technology Cooperation Centre, Wenzhou-Kean University, 88 Daxue Road, Ouhai, Wenzhou, Zhejiang 325060 China; 3 Wenzhou Municipal Key Laboratory for Applied Biomedical and Biopharmaceutical Informatics, 88 Daxue Road, Ouhai, Wenzhou, Zhejiang 325060 China; 4 China Biodiversity Conservation and Green Development Foundation (CBCGDF), Empark International Apartment, No. 69, Banding Road, Haidian District, Beijing, China; 5 Dorothy and George Hennings College of Science, Mathematics and Technology, Kean University, 1000 Morris Ave, Union, NJ 07083 USA

**Keywords:** Animal breeding, Functional genomics, Gene expression, Genetic association study, Immunogenetics, Sequencing

## Abstract

Protection of the Critically Endangered East Asian Pangolin species is hampered by the vulnerability of captive individuals to infection. Studies have previously shown the pangolin to have a unique pseudogenisation of many immunity genes (including *IFNE*, *IFIH1*, *cGAS*, *STING*, *TLR5*, and *TLR11*), and we suspected that these losses could account for this vulnerability. Here we used RNA-Seq data to show the effect of these gene losses on the transcriptional response to a viral skin infection in a deceased pangolin. This virus is very closely related to the one causing the current COVID-19 pandemic in the human population (SARS-CoV2), and we found the most upregulated pathway was the same one previously identified in the lungs of SARS-CoV2-infected humans. As predicted, we found that the pathways downstream of the lost genes were not upregulated. For example, the pseudogenised interferon epsilon (*IFNE*) is known to be particularly important in epithelial immunity, and we show that interferon-related responses were not upregulated in the infected pangolin skin. We suggest that the pangolin’s innate gene pseudogenisation is indeed likely to be responsible for the animal’s vulnerability to infection.

## Introduction

Pangolins are 30–100 cm long ant- and termite-feeders found in Africa (two *Phataginus* spp. and two *Smutsia* spp.) and Asia (four *Manis* spp.)^1^. They have converged morphologically with unrelated South American anteaters. All three species occur in East Asia [Chinese pangolin (*M. pentadactyla*), Philippine pangolin (*M. culionensis*) and Malayan pangolin (*M. javanica*)] are *Critically Endangered*, with the Indian pangolin (*M. crassicaudata*) being *Endangered*^2^. The four African species are either *Endangered* or *Vulnerable*^2^ and all eight species have declining populations^2^. One of the main threats to the conservation of pangolins is poaching for body parts used in traditional medical remedies^3^. Pangolins are especially important to conserve because of their phylogenetic uniqueness: they are the only extant members of their order (Pholidota).

One of the main pangolin conservation challenges is that captive pangolins usually die from infection^3^. This makes it very difficult to maintain captive breeding programs or return rescued animals to the wild. In response to this problem, rigorous hygiene protocols have enabled us to establish a captive Malayan pangolin population up to the third filial generation in China^4^. This vulnerability to infection is possibly due to the pseudogenisation of immune system genes in the pangolin genome, including Interferon Epsilon (*IFNE*)^1^, Interferon-Induced with Helicase C domain 1 (*IFIH1*, also known as *MDA5*, a cytoplasmic RNA sensor that helps initiates the innate immune response to viral infection)^5^, cyclic GMP-AMP Synthase (*cGAS*)^6^, Stimulator of Interferon Genes (*STING*, the interaction partner of cGAS)^6^, Toll-Like Receptor 5 (*TLR5*)^7^, and also likely Toll-Like Receptor 11 (*TLR11*)^7^.

Owing to its rarity and protected status, Malayan pangolin specimens that can be examined are difficult to obtain. We investigated a possible infection in a Malayan pangolin that was seized by customs in the Guangdong province of China, which subsequently died^8^. We previously reported that this specimen’s brain (cerebrum and cerebellum) and lungs were infected by a coronavirus (pCoV) closely related to SARS-CoV-2^9,10^, the cause of the current human pandemic COVID-19^8^. Indeed, at the start of the pandemic, it was proposed that the Malayan pangolin was the intermediate host of bat-to-human transmission of SARS-CoV-2^11,12^; however, subsequent sequence analysis has shown this not to be the case^13^.

In this study, we selected pangolin skin tissue for RNA-Seq analysis because we had access to an appropriate control sample (uninfected/healthy skin), unlike for the lung where no suitable control was available. This choice was driven by the aim to investigate the transcriptional antiviral response in pangolin skin, particularly in the context of *IFNE*-deficiency, which is expressed in both skin and lungs. Notably, while we compared coronavirus-infected pangolin skin with healthy pangolin skin for differentially expressed genes (DEGs), we also compared these DEGs with those identified in coronavirus-infected human lungs. This was due to the absence of a corresponding dataset for coronavirus-infected human skin. Our comparative analysis aimed to cross-validate our findings in pangolin skin, presuming some similarity in immune responses between human lungs and pangolin skin as both are mammals. Understanding this response in the skin is crucial for comprehending pangolin’s immune response to viral infections, given the species’ unique immunity characteristics.

Crucially, we also investigated the expression of endogenous retrovirus (ERV) genes in the context of pCoV infection. Given the reported role of ERVs as modulators of innate immunity and their support for the antiviral immune response through various mechanisms^14–16^, our study also aims to understand how ERV gene expression in pangolins responds to pCoV, especially considering their unique immune characteristics, including *IFNE* deficiency.

## Materials and methods

### Animal ethics approval

This work was approved (reference number: GF(2019)BASE08) by the Biology and Science Ethics Committee of the China Biodiversity Conservation and Green Development Foundation (CBCGDF). All methods were carried out in accordance with relevant guidelines and regulations. The skin sample came from the pangolin named ‘Dahu’. The source from where and how we obtained the pangolin samples for this study were detailed in our previous publication^8^.

### Sample preparation, RNA extraction, and sequencing

Total RNA of the skin sample with pCoV infection was extracted using Qiagen RNeasy Mini Kit (QIAGEN, Netherlands). A limitation of this study is that we only have one pCoV infected skin sample since it is extremely difficult to find the CoV-infected pangolin samples particularly they are endangered species. Additionally, due to safety concerns during the COVID-19 pandemic, all samples have been destroyed, rendering it impossible to validate the RNA-Seq results using the same samples with RT-qPCR. However, the quality and quantity of the RNA were examined using Agilent 2100 Bioanalyzer (Agilent Technologies, CA, USA), NanoDrop (Thermo Fisher Scientific, MA, USA), and 1% agarose gel. Then, 1 μg of total RNA with an RNA integrity number (RIN) above 6.5 was used for library preparation. mRNA with poly-A tail was isolated using Poly-A mRNA Magnetic Isolation Module or rRNA removal kit (New England Biolabs, MA, USA). Then, the isolated mRNA was fragmented, and priming was performed using random primers and First-Strand Synthesis Reaction Buffer (New England Biolabs, MA, USA). First-strand cDNA was synthesised using ProtoScript II Reverse Transcriptase (New England Biolabs, MA, USA) and the second-strand cDNA was synthesised using Second Strand Synthesis Enzyme Mix (New England Biolabs, MA, USA). Then, the purified double-stranded cDNA was end-repaired using End Prep Enzyme Mix (New England Biolabs, MA, USA). A dA-tail was added on each end of cDNA to ligate adapters. Size selection of adaptor-ligated cDNA was then performed using beads, and fragments of around 420 bp (with the approximate insert size of 300 bp) were retained.

The sample was then amplified by polymerase chain reaction (PCR) for 13 cycles using P5 and P7 primers, with both primers carrying sequences which can attach to flow cell to perform bridge amplification and P7 primer carrying a six-base index allowing for multiplexing. The PCR products were purified, before being validated using Qsep100 (Bioptic, Taiwan, China) and quantified by Qubit3.0 Fluorometer (Invitrogen, CA, USA). Libraries with different indices were multiplexed and loaded on an Illumina HiSeq instrument according to manufacturer’s instructions (Illumina, CA, USA). Sequencing was carried out using 2 × 150 bp paired-end mode on an Illumina HiSeq instrument. The sequencing yielded approximately 22 million paired end reads.

### Raw data pre-processing and mapping

Qualities of raw reads in FASTQ format were checked using FastQC v0.11.9^17^. Low-quality bases with Phred score lower than 20, adapter sequences, and PCR primers were trimmed using Cutadapt v1.9.1^18^. 21,956,237 pairs of reads of length 150 bp generated from the pangolin skin passed the quality check.

The healthy Malayan pangolin skin sample was downloaded from our previous study (project accession PRJNA283328; run SRR3923846) and quality checked. Malayan pangolin (*Manis javanica*) primary genome assembly (ManJav1.0; accession: GCF_001685135.1) and the corresponding GTF annotation were downloaded from National Centre for Biotechnology Information (NCBI). 24,805,754 pairs of reads of length 150 bp from normal pangolin skin passed the quality check. Then, the primary genome assembly was indexed using Bowtie2 v2.4.2^19^ and the trimmed reads from the healthy and Dahu skin samples were mapped to the genome using TopHat v2.1.1^20–22^. The overall mapping rates for the pangolin skin and healthy pangolin skin were 76.2% and 67.4%, respectively.

We also downloaded the data of human lung biopsies from the study of Blanco-Melo et al.^23^ (project accession PRJNA615032), including healthy human lung biopsies from a 72-year-old male and a 60-year-old female (runs SRR11517725, SRR11517726, SRR11517727, SRR11517728, SRR11517729, SRR11517730, SRR11517731, and SRR11517732), and SARS-CoV-2 infected lung biopsies from a deceased 74-year-old male (runs SRR11517733, SRR11517734, SRR11517735, SRR11517736, SRR11517737, SRR11517738, SRR11517739, and SRR11517740) (Supplementary Table S1). The reads were quality-checked, indexed, and mapped using the same approaches as above. Technical replicates were merged before being analysed. The overall mapping rate for healthy and human lung samples were 87.7% and 85.0%, respectively, and for SARS-CoV-2 infected human lung samples, the overall mapping rates were 61.0% and 71.0%, respectively.

### Examination of the presence of SARS-CoV-2 RNA

All reads were mapped to pCoV genome to confirm the presence of pCoV RNA in the pangolin skin transcriptome. The pCoV genome was obtained from Xiao et al.^9^. To perform gapped local read alignment, we indexed the trimmed reads using Burrows-Wheeler transformation (BWA) aligner v0.7.17-r1188^24^ and mapped using BWA MEM with the option -T 45. For visualisation purposes, the genomes and mapped reads (sorted BAM files) were indexed using SAMtools v1.11^25,26^, and visualised using Integrative Genomics Viewer (IGV) v2.4.9^27–29^.

### Phylogenetic analysis

The pCoV partial genome or gene sequences from the mapped reads were extracted from IGV v2.4.9^27–29^. Phylogenetic trees were constructed using MEGA-X^30^. Sequences were initially aligned using Multiple Sequence Comparison by Log-Expectation (MUSCLE) aligner^31^. The alignments were manually curated to ensure accuracy. Maximum-likelihood trees were inferred using the Tamura-Nei DNA substitution model and nodal support was estimated using 1,000 bootstrap replicates.

### Differential expression (DE) analysis

The read counts and normalised Fragments Per Kilobase Million (FPKM) for each gene were generated according to the Cufflinks pipeline^32^. The genes of FPKM less than one were considered as low expression or noise and being filtered. The remaining genes were considered as up-regulated DEGs if log_2_ fold change (FC) were higher than one, and down-regulated DEGs if log_2_ FC were lower than one (coronavirus versus control). For human samples, we downloaded the raw data from Blanco-Melo et al.^23^ and processed them using the same approach for accurate comparison. To compare the human gene expression with pangolin, we generated the normalised read counts for each human sample using the same approaches as above.

### Functional enrichment analysis

Gene set enrichment analysis (GSEA) and over-representation analysis (ORA) were performed using clusterProfiler v3.18.0^33^. GSEA was done using all the pangolin genes or human genes using fgsea method^34^.

We performed ORA using pangolin skin-specific or human lung-specific DEGs, compared against all the genes in pangolin or human (as background). Gene sets used in ORA and GSEA were based on human, including gene ontology (GO) biological process (BP) gene sets^35^ queried using AnnotationHub v2.22.0^36^ and Kyoto Encyclopedia of Genes and Genomes (KEGG) pathway gene sets^37^. For ORA, we defined an enrichment score for each enriched term as below

$$\frac{{F}_{in}/{F}_{all}}{{B}_{in}/{B}_{all}},$$

where ${F}_{in}$ and ${B}_{in}$ are number of genes belonging to the term in the foreground (test) gene set and background gene set, and ${F}_{all}$ and ${B}_{all}$ are the number of all the genes in the foreground (test) and background gene set. The results with false discovery rate (FDR) adjusted *p*-values lower than 0.05 were considered as significant.

### Analysing the expression of ERV genes

3,162 common viral proteins in the UniProt database were downloaded using the criteria: "Viruses [10239] " (name:helicase OR gene:gag OR gene:c OR gene:pol OR gene:env OR gene:tat OR gene:s OR gene:rev OR gene:rep OR name:polymerase OR gene:nef) AND reviewed:yes. The endogenous viral genes were explored using the TBLASTN^38^ output by querying common viral proteins against unmasked host genome. We retained hits with more than 40% identity, e-value lower than 1 × 10^–6^, and bitscore higher than 60. Then, among the retained hits, we selected one representative sequence among overlapping results by selecting the highest completeness hit. We removed all non-retroviruses and genes that have overlap coordinates with known exons. The filtered results were considered as ERV genes in the pangolin genome.

## Results

### The presence of pCoV subgenomic mRNA in the skin

**Figure 1:**
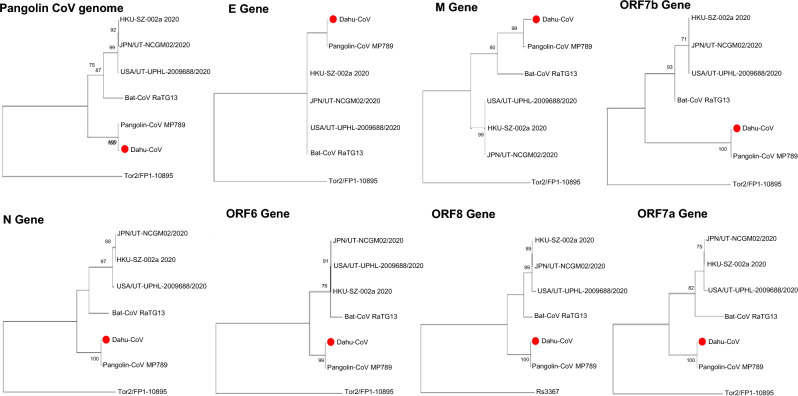
Phylogenetic trees of the coronavirus RNA and gene sequences from the Malayan pangolin’s skin. Phylogenetic trees were generated using the maximum likelihood, with 1000 bootstrap replicates. Nodes with bootstrap support values of 70 or greater are indicated.

To confirm that this specimen’s skin was infected by pCoV (in addition to its lungs and brains^8^), we searched for viral sequences among the transcriptome data. A total of 193 reads mapped to the reference pCoV genome (Fig. S1 in Supplement 1). The distribution of these mapped reads was consistent with the corresponding locations of the subgenomic mRNAs^39^. We also observed an individual read that spanned precisely the splicing sites: this read was 150 bp in length and its 5′ 47 bp mapped to the 5’ region of the pCoV genome, while its 3′ 103 bp mapped to the 3’ region of the same genome. This read indicates that the CoV RNA has been processed in the host cell^40^. We consistently detected the N gene (which is used in diagnostic human SARS-CoV-2 testing) in all the tested organs using qRT-PCR^8^, including the skin. To confirm the identity of the viral RNA in the skin, we compared consensus sequences from our mapped reads with CoV from other species (Fig. 1; Supplementary Table S2). We observed that the pCoV genome and genes from the specimen’s skin were almost identical (sequence identity = 99.2–100%) to the counterparts from another pangolin, Guangdong pCoV isolate MP789^41^, confirming the presence of pCoV RNA in the current pangolin’s skin.

### Comparative analysis of transcription in Malayan pangolin skin and human lungs

Our comparative analysis of transcription focused on Malayan pangolin skin and human lungs. It is crucial to clarify that this comparison was not due to direct similarity between these tissues, but rather because of the availability of comprehensive data on DEGs in coronavirus-infected human lungs, which contrasts with the absence of such data for coronavirus-infected human skin. We leveraged this comparison as part of our cross-validation strategy for the DEGs identified in pangolin skin, operating under the assumption that certain similarities in immune response exist between human lungs and pangolin skin, given their shared mammalian traits.

**Figure 2:**
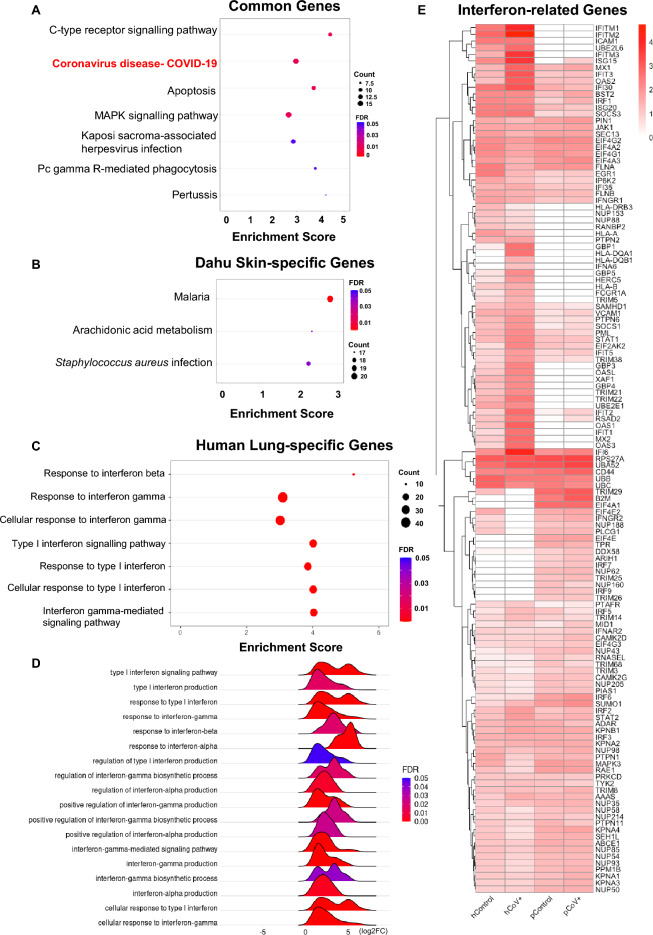
Comparative analysis and interferon-related responses in Malayan pangolin (*Manis javanica*) skin and human lung tissue. (**A**) Enriched pathways in common differentially expressed genes (DEGs) between the Malayan pangolin skin and human lung. (**B**) Enriched pathways in Malayan pangolin skin-specific DEGs. (**C**) Interferon-specific responses significantly enriched in human-specific DEGs. (**D**) Significant interferon-related terms from the human gene set enrichment analysis (GSEA) results. (**E**) Interferon pathway related gene expressions in healthy human lungs (hControl), SARS-CoV-2 infected human lungs (hCoV +), healthy pangolin skin (pControl), and pCoV infected pangolin skin (pCoV +). Gene expression in Fragments Per Kilobase of transcript per Million mapped reads (FPKM) were log_10_ transformed and only expressed genes are shown. *GO* gene ontology, *BP* biological process, *FDR* false discovery rate adjusted *p*-value, *KEGG* Kyoto Encyclopedia of Genes and Genomes.

Here, we identified 3201 DEGs (1810 upregulated and 1391 down-regulated) in the Malayan pangolin specimen’s skin (Supplementary Table S3). To validate our data, we investigated the differences and similarities of host responses between the Malayan pangolin’s skin and the human lungs by comparing DEG lists between our study and an external human lung study (Supplementary Table S1)^23^. Our comparative analysis revealed 366 DEGs shared between species (i.e., ‘common DEGs’ below), 2835 Malayan pangolin skin-specific DEGs, and 1527 human lung-specific DEGs. As anticipated, the common DEGs were enriched in coronavirus diseases-COVID-19 pathway, followed by MAPK signalling pathway, apoptosis, C-type lectin receptor signalling pathway and Kaposi sarcoma-associated herpesvirus infection. These findings are consistent with the pCoV infection in the Malayan pangolin’s skin (Fig. 2A).

Consistent with the pangolin’s unique immune characteristics^1,5–7^, especially the loss of IFNE which plays an important antiviral role in epithelial cells^42–48^, interferon responses (e.g., interferon-gamma production) were significantly enriched and upregulated in human lung-specific DEGs (Fig. 2C,D; Supplementary Table S5), but not in the Malayan pangolin skin-specific DEGs (Supplementary Table S6). We also downloaded genes related to interferon signalling pathways in Reactome pathway database^49,50^ and found 131 genes were expressed in the Malayan pangolin’s skin and/or human lungs (Fig. 2E; Supplementary Table S7). Among them, 102 genes were expressed in human lungs while 88 genes were expressed in the Malayan pangolin skin (Fig. 2E). We found 42 genes (37 upregulated and five down-regulated) that were differentially expressed in human lungs, but none of them was differentially expressed in the Malayan pangolin skin.

In our comparative analysis, we found three enriched pathways in the Malayan pangolin skin-specific DEGs (Fig. 2B): malaria and *Staphylococcus aureus* infection pathways were upregulated while arachidonic acid (AA) metabolism pathways were down-regulated. The malaria pathway was also upregulated in human lungs. Malaria pathway is commonly upregulated after SARS-CoV-2 infection^51^, and anti-malarial drugs have shown effects on inhibiting SARS-CoV-2 replication^52^. The downregulation of the AA metabolism pathways in the Malayan pangolin skin-specific DEGs (Fig. 2B) indicates that these pathways were suppressed by pCoV infection. It is known that AA pathways have inhibitory effects on coronavirus replication, suggesting that lipid metabolism could be a druggable target of coronavirus-infected patients^53^. Therefore, pCoV might suppress AA metabolism pathway in pangolin skin to benefit its replication.

**Figure 3:**
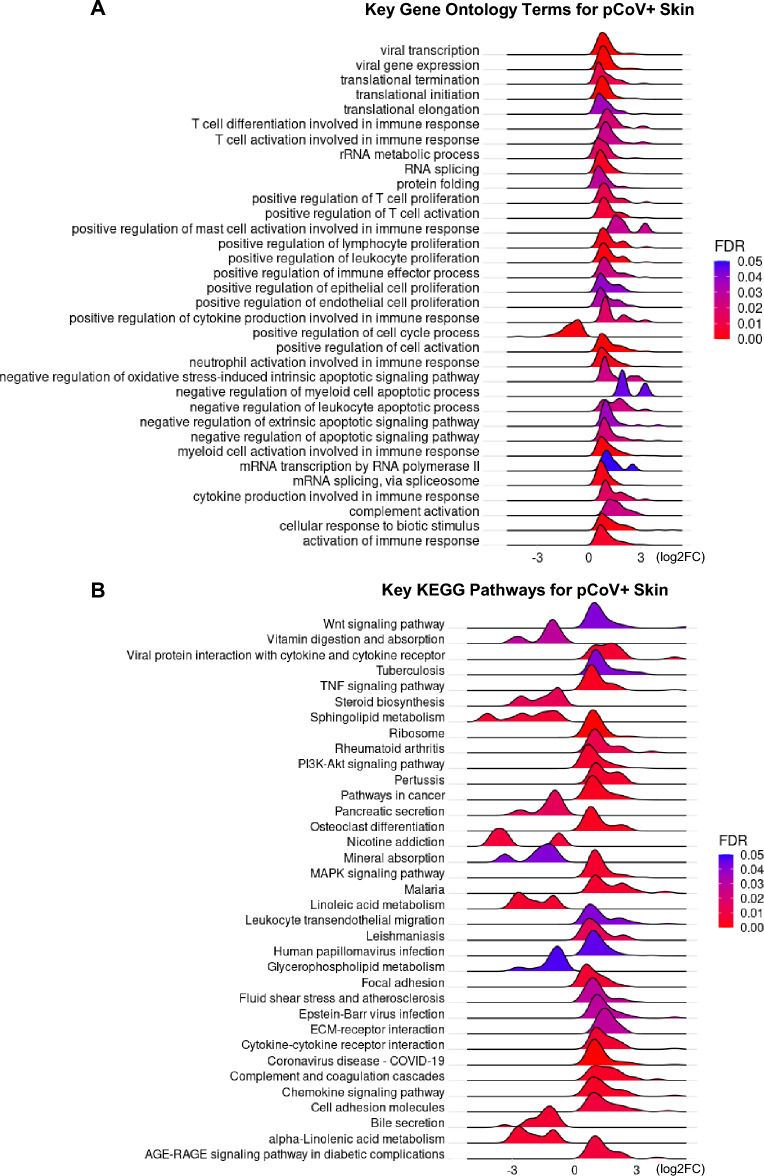
Gene set enrichment analysis of pangolin genes. Ridge plots of (**A**) key Gene Ontology biological process (GO BP) terms and (**B**) Kyoto Encyclopaedia of Genes and Genomes (KEGG) pathways. *FDR* false discovery rate adjusted *p*-value.

Consistent with the results observed in human COVID-19 patients^51^, we found that a range of other terms associated with immunity processes were significantly enriched and upregulated, such as terms related to non-interferon cytokine production, T cell, neutrophil, myeloid cell, and mast cell differentiation and activation (Fig. 3A). Apoptotic signalling pathways were negatively regulated, especially apoptotic processes of immune cells, such as myeloid cells and leukocytes. Also, rRNA metabolic process, RNA splicing, mRNA transcription, translational processes, and protein folding were also upregulated. Specifically, we found that genes associated with viral transcription and viral gene expression were upregulated. Other terms, including complements and cellular response to biotic stimulus, were upregulated, while cell cycle processes were downregulated.

As expected, we found that the coronavirus disease-COVID-19 pathway was the most significant upregulated pathway (Fig. 3B; Supplementary Table S4). Ribosomal proteins were upregulated (Fig. 3B; Supplementary Table S4) as CoV needs ribosome frameshifting to translate and replicate^54,55^. It has been shown that the nonstructural protein 1 of the SARS-CoV-2 is a major virulence factor that interferes with host RNA translation by binding to 40S ribosomal subunit^56,57^. Li summarised the functional relationships between host ribosomal proteins and viral infection^58^ and suggested most interactions are beneficial for viral protein translation and replication. We found that ribosomal proteins that are crucial for viral infection were upregulated in the Malayan pangolin skin (Supplementary Fig. S2), such as RPL3^59^, RPL18^60–63^ and RPL24^64^. Moreover, we found that RPL9 and RPL22, which help virus particle assembly and viral gene expression, were upregulated in the Malayan pangolin skin, but not human lungs (Supplementary Fig. S2B), probably a new strategy to promote its replication in pangolins^65,66^.

Other upregulated pathways include pathways in cancer, TNF signalling pathway, complement and coagulation cascades, chemokine signalling pathway, immunity pathway, and viral interaction/infection related pathways (Fig. 3B; Supplementary Table S4). Furthermore, the DEGs were down-regulated in pathways associated with linoleic acid and lipid metabolism (Fig. 3B; Supplementary Table S4). Again, these host responses are consistent with the core pathways identified in the human SARS-CoV-2 infection^51^.

### Responses of endogenous retrovirus (ERV) gene expression in the pCoV skin infection

In our analysis of the Malayan pangolin skin infected with pCoV, we paid particular attention to the expression of ERV genes. This focus stems from various studies highlighting ERVs as potential modulators of the innate immune system and their capability to support antiviral responses through different mechanisms^14–16^. Our goal was to elucidate how ERV gene expression in pangolins is influenced by CoV infection, providing insights into the complex interplay between endogenous retroviruses and viral pathogens in a species with unique immunological traits.

**Figure 4:**
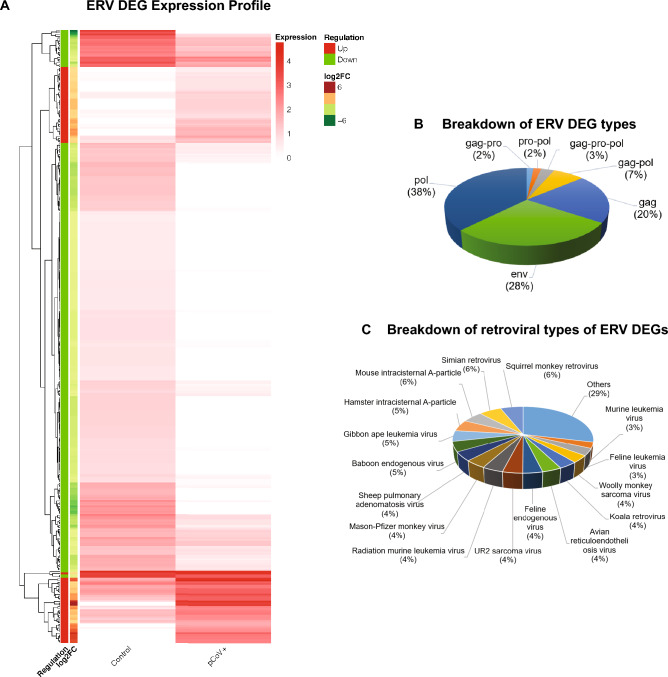
Differentially expressed endogenous retrovirus (ERV) gene composition and expression profiles. (**A**) ERV differential expressed gene (DEG) expressions. The 'regulation' indicates if the gene is up- or down-regulated in the pCoV infected pangolin skin. The 'log2FC' indicates the log2 fold change of the corresponding gene, and positive values represent up-regulation in the pCoV infected pangolin skin. ‘Regulation’ and ‘log2FC’ correspond to the pCoV infected skin compared to uninfected skin. (**B**) Breakdown of ERV DEG types. (**C**) Breakdown of retroviral types of ERV DEGs. 'pol' refers to genes coding for replication enzymes like reverse transcriptase; 'env' denotes envelope protein genes, crucial for cell entry; 'gag' represents genes for core structural proteins. Combinations like 'gag-pro' (structural proteins and protease), 'pro-pol' (protease and polymerase), 'gag-pro-pol' (structural proteins, protease, and polymerase), and 'gag-pol' (structural proteins and polymerase) indicate fused gene sequences with multiple functionalities essential for viral replication and assembly. The gene expressions in Fragments Per Kilobase of transcript per Million mapped reads (FPKM) were log_2_ transformed before being divided by two. Retroviral names are taken from the closest match in our library of viral gene sequences.

Here, we identified 6,076 ERV genes by screening 3,162 known viral proteins from Swiss-Prot across the pangolin genome (Supplementary Table S8). We found 466 genes expressed in infected and/or non-infected pangolin skin, of which 348 were differentially expressed (81 upregulated versus 267 downregulated) (Fig. 4A; Supplementary Table S9), suggesting that the exogenous pCoV might suppress the expression of ERV genes after infection to benefit its replication. We found that most of the ERV DEGs were env (43%), pol (31%), and gag (16%) (Fig. 4B; Supplementary Table S9), while the compositions in the genome were 28%, 38%, and 20%, respectively (Supplementary Table S8). The most abundant group of ERV DEGs were most closely related to mouse intracisternal A-particle (IAP) viruses (Fig. 4C**; **Supplementary Table S9). Mouse and Hamster IAP viruses make up 19% of the ERV DEGs, while only 11% of the IAP sequences are found in the pangolin genome (Supplementary Table S8), which may suggest the importance of IAPs in CoV infection and their possible interaction with the virus.

## Discussion

In this study, pCoV RNA was found in the Malayan pangolin skin and this was almost identical to pCoV MP789 from the Guangdong pangolin. Both the current Malayan pangolin and the Guangdong pangolin MP789 were seized by Guangdong anti-smuggling bureaus after being smuggled into China^8^ and kept together at Guangdong wildlife rescue centre. Therefore, it is possible that both pCoVs originated from the same source^8^.

Pangolins have weakened immune systems due to the pseudogenisation of immunity-related genes, such as *IFNE*, *IFIH1*, *cGAS*, *STING*, *TLR5*, and *TLR11*^1,6,7^. There have been many attempts to maintain and breed individuals of this *Critically Endangered* species in captivity in the past, but mostly with little success due to infection^3,67^. Therefore, to maintain pangolins in captivity, it is critical to provide a clean environment to reduce infection. Recently, for the first time, we have successfully bred a captive Malayan pangolin population to the third filial generation by keeping the environment, food and water as hygienic as possible under proper husbandry^4^.

The mechanism of pCoV entry into Malayan pangolin skin cells remains unclear. While the low or undetectable expression of ACE2 in this specimen's skin aligns with the observed low interferon levels, it does not definitively preclude its presence or functionality as a viral entry receptor. This conclusion is drawn considering that ACE2 expression, even in healthy human lungs where it serves as the primary receptor for SARS-CoV-2, is typically low^68^. Additionally, our analysis suggests the potential involvement of alternative receptors, such as DPP4, which has been identified in the infected pangolin skin and is recognised as a potential binding target for SARS-CoV-2^69^. This consideration is particularly relevant given the distinct expression profiles of pangolin keratinocytes compared to humans. Therefore, while our findings suggest a potential role for ACE2 in pCoV infection, the possibility of alternative or supplementary entry mechanisms, such as through DPP4, cannot be ruled out. Considering the situation in humans, clinical and histopathological studies of COVID-19 patients reported some dermatologic manifestations such as petechiae (a rash and haemorrhagic dot-like areas)^70–73^, and it has been suggested that angiotensin-converting enzyme 2 (ACE2)—used by SARS-CoV-2 to enter the host—can be highly expressed in keratinocytes^74,75^. Therefore, we cannot rule out the possibility that the skin of these patients was indeed infected by SARS-CoV-2 and novel mechanisms may exist to assist CoV in infecting the skin. Our functional enrichment analyses are generally consistent with the results observed in human patients with SARS-CoV-2 infection^51^. Furthermore, cell cycle processes were suppressed in pangolin skin. It has been shown that CoV can arrest cell cycle to boost viral replication efficiency^76^ through mechanisms such as regulating through cyclin-CDK complex^77^, p53-dependent pathway^78^, N protein of coronavirus^79^, and directly interacting with host cell cycle proteins^80^. At the pathway level, our analysis showed that the COVID-19 pathway, immunity and inflammation (except for IFN-related pathways), cell proliferation, and coagulation pathways were the most significant upregulated pathways in the Malayan pangolin’s skin^51^. In the CoV-infected pangolin skin, the interferon-specific pathways were not enriched, and the expressions of many interferon pathway-related genes were undetectable and/or not significantly differentially expressed. Therefore, the IFNE-mediated pathways, including interferon-stimulated gene responses, are unlikely to be activated or upregulated in the naturally IFNE- and IFIH1-deficient pangolins upon CoV infection^1^.

In this study, we examined the expression of ERV genes in the context of pCoV infection in pangolin skin. ERVs are known to be significant modulators of the innate immune system and can support antiviral responses through various mechanisms^14–16^. Our analysis aimed to understand how ERV gene expression in pangolins responds to pCoV, given their potential role in enhancing antiviral defense. In healthy pangolin skin, many ERV genes were expressed, indicating their biological significance. We believe that these ERV genes are beneficial to the host, such as to boost the host’s immunity^81,82^, which is especially important in IFNE-deficient pangolins. Our data showed that most of the ERV genes were downregulated after being infected, leading to our speculation that pCoV might directly or indirectly suppress the ERV genes to benefit its own proliferation. It is important to note that skin tissue was specifically chosen for RNA-Seq analysis due to the availability of an appropriate control sample, unlike the lung where no suitable control was available. Our investigation into skin tissue is significant, as it provides insights into the transcriptional antiviral response of pangolins, especially given their unique immune characteristics and the *IFNE*-deficiency which is expressed in both skin and lungs. This focus allows for a comprehensive understanding of the species' response to viral infections and contributes to our broader knowledge of pangolin immunity.

A possible cause of observation of replication of pCoV in the skin is contamination by pCoV-infected blood. Also, a limitation of this study is that our observations are only based on one sample due to the fact that Malayan pangolin is a *Critically Endangered* species found in Southeast Asia and difficult access, making it extremely tough to study them. Therefore, it would be useful to validate these results using more samples (whenever it is available) in the future.

## Conclusion

We observed pathway dysregulation consistent with CoV infection of an organism lacking multiple immunity-related genes. We also report the presence of replicating virus in the skin (proven by the presence of pCoV subgenomic/spliced mRNAs which only occur in infected cells) and transcriptomic hallmarks of the host response to CoV infection. This study highlights the unique transcriptional response of pangolins to viral infections, which is impacted by the pseudogenisation of key immunity-related genes. Also, it underscores the value of studying pangolin antiviral responses to enhance our understanding of similar processes in humans.

### Supplementary Information

Supplementary Figure S1.

Supplementary Figure S2.

Supplementary Table S1.

Supplementary Table S2.

Supplementary Table S3.

Supplementary Table S4.

Supplementary Table S5.

Supplementary Table S6.

Supplementary Table S7.

Supplementary Table S8.

Supplementary Table S9.

Supplementary Legends.

## Data Availability

The sequencing data have been deposited in the CNSA (https://db.cngb.org/cnsa/) of CNGBdb with accession number CNP0001573. Supporting data is included in the published paper and/or the additional information. For any additional information, request can be sent to the corresponding author at cwoh@wku.edu.cn.
