# Supplementary figures and images for: An RNA-Seq analysis of coronavirus in the skin of the Pangolin
Source: Sci Rep. 2024 Jan 9;14:910. doi: 10.1038/s41598-024-51261-x (PMC10776870; doi:10.1038/s41598-024-51261-x)

Mapping of pCoV infected pangolin skin RNA-seq reads to pCoV genome

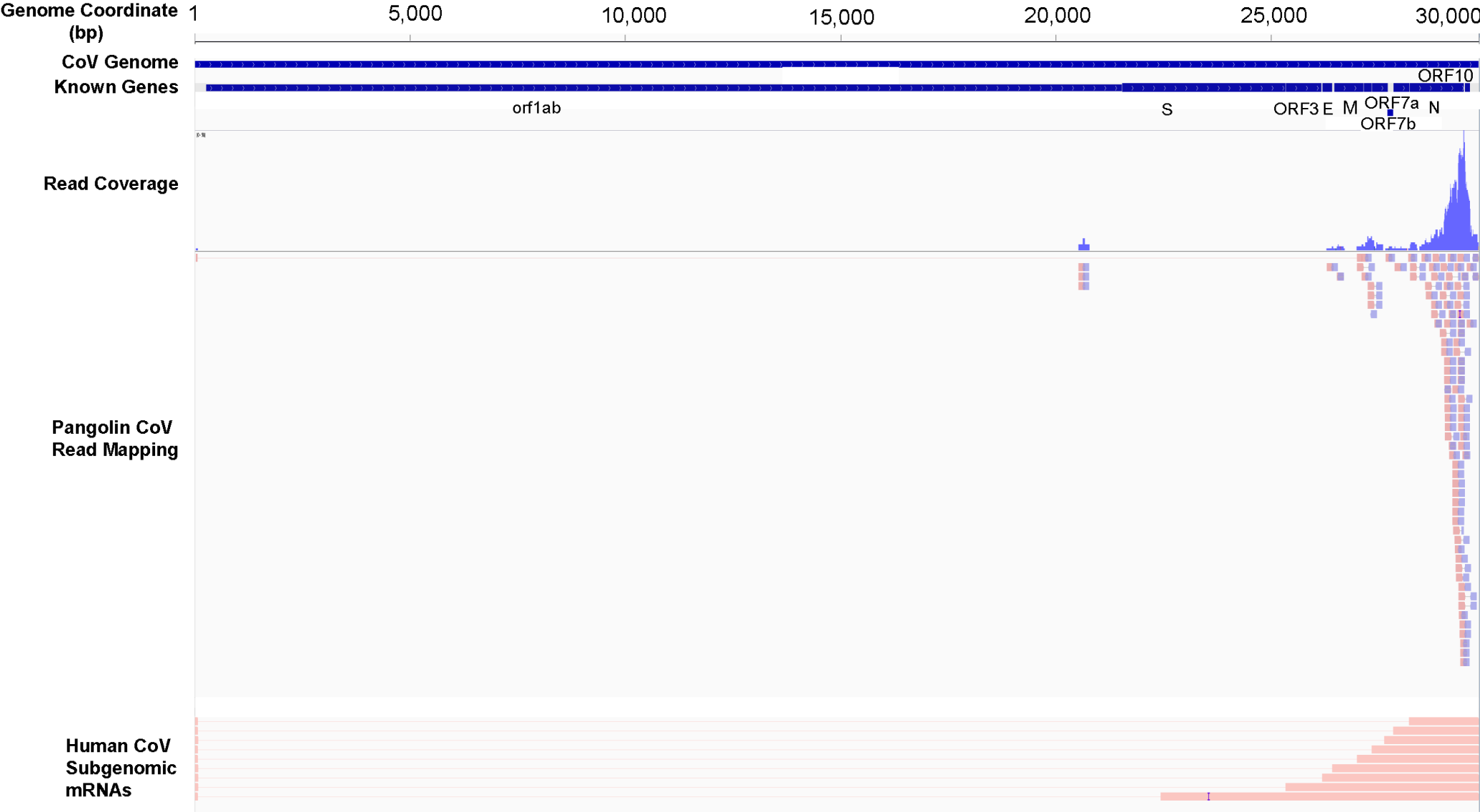

Supplement: Supplementary file 1 — Supplementary Figure S1. [file 41598_2024_51261_MOESM1_ESM.pdf]

# A

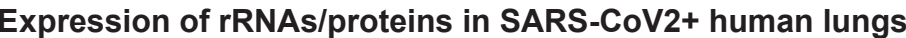

## B

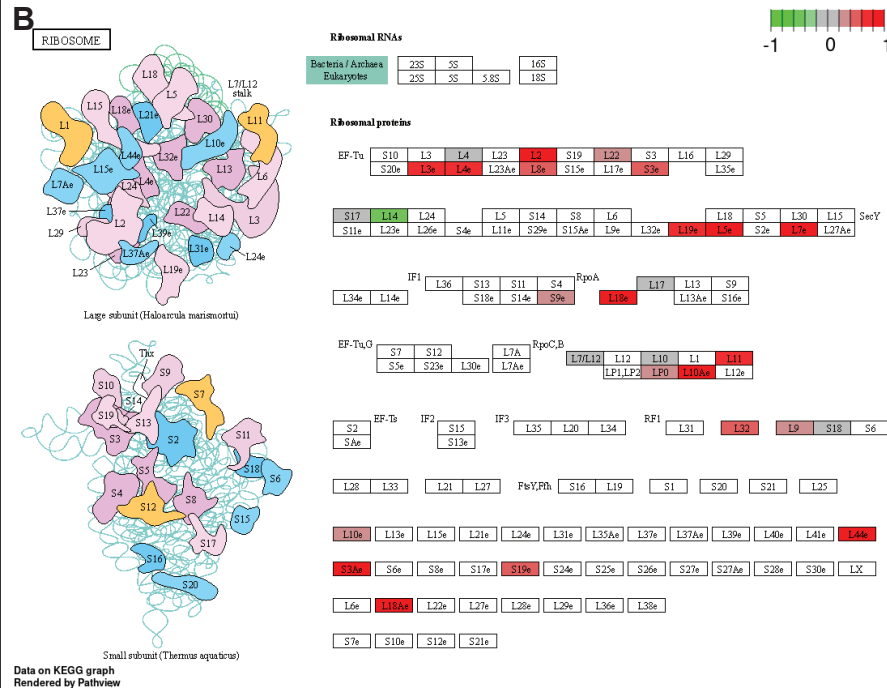

Supplement: Supplementary file 2 — Supplementary Figure S2. [file 41598_2024_51261_MOESM2_ESM.pdf]
