# Supplementary material for: An RNA-Seq analysis of coronavirus in the skin of the Pangolin
Source: Sci Rep. 2024 Jan 9;14:910. doi: 10.1038/s41598-024-51261-x (PMC10776870; doi:10.1038/s41598-024-51261-x)
Supplement: Supplementary file 12 — Supplementary Legends. [file 41598_2024_51261_MOESM12_ESM.docx]

**Figure S1. Mapping of Dahu skin RNA-seq reads to pCoV genome.**

**Figure S2. Expression of ribosomal RNAs and proteins in (A) Dahu skin and (B) human lungs.** Red indicates upregulated and green indicates downregulated. The number on the top-right corner indicates log2 fold change.
